# Targeted anti-staphylococcal therapy with endolysins in atopic dermatitis and the effect on steroid use, disease severity and the microbiome: study protocol for a randomized controlled trial (MAAS trial)

**DOI:** 10.1186/s13063-017-2118-x

**Published:** 2017-08-31

**Authors:** Joan Totté, Jill de Wit, Luba Pardo, Frank Schuren, Martijn van Doorn, Suzanne Pasmans

**Affiliations:** 1 Department of Dermatology, Erasmus MC University Medical Center Rotterdam, Wytemaweg 80, 3015 CN Rotterdam, The Netherlands; 2 TNO, Microbiology and Systems Biology Group, Utrechtseweg 48, PO Box 360, 3700 AJ Zeist, The Netherlands; 3 Department of Pediatric Dermatology, Sophia Children’s Hospital, Erasmus MC University Medical Center Rotterdam, Wytemaweg 80, 3015 CN Rotterdam, The Netherlands

**Keywords:** Atopic dermatitis, Eczema, *Staphylococcus aureus*, Endolysins, Lysins, Staphefekt, Microbiome

## Abstract

**Background:**

Atopic dermatitis (AD) is associated with reduced skin microbial diversity and overgrowth of *Staphylococcus (S.) aureus*. However, the importance of *S. aureus* colonisation in the complex pathogenesis remains unclear and studies on the effect of anti-staphylococcal therapy in non-infected AD show contradictory results. Long-term interventions against *S. aureus* might be needed to restore the microbial balance, but carry the risk of bacterial resistance induction. Staphefekt, an engineered bacteriophage endolysin, specifically kills *S. aureus* leaving other skin commensals unharmed. Bacterial resistance towards endolysins has not been reported, nor is it expected, which allows us to study its effect as long-term anti-staphylococcal treatment in non-infected AD.

**Methods:**

This is a multi-centre, placebo-controlled, double-blinded and randomized superiority trial with a parallel group design. A total of 100 participants, aged 18 years or older, diagnosed with moderate to severe AD and using a topical corticosteroid in the weeks before enrolment are included in the study. The study is executed in the Erasmus MC University Medical Centre Rotterdam in collaboration with the Havenziekenhuis Rotterdam. After a 2-week run-in period to standardize the corticosteroid use with triamcinolone acetonide 0.1% cream, participants will be randomized to either treatment with Staphefekt in a cetomacrogol-based cream or a placebo for 12 weeks, followed by an 8-week follow-up period. The primary objective is to assess the difference in the need for corticosteroid co-therapy between the Staphefekt and the placebo group, measuring the number of days per week of corticosteroid cream (triamcinolone) use. Secondary outcomes include the difference in use of corticosteroid cream measured in grams, differences in clinical efficacy, quality of life (QoL), microbial composition (includi23ng *S. aureus*) between the Staphefekt and the placebo group, and the safety and tolerability.

**Discussion:**

The results of this trial will provide data about the effect of long-term anti-staphylococcal therapy with Staphefekt on corticosteroid use, clinical symptoms and QoL in patients with moderate to severe AD. Additional data about growth characteristics of the skin microbiome, including *S. aureus*, will give insight into the role of the microbiome as a factor in the pathophysiology of AD.

**Trial registration:**

ClinicalTrials.gov, NCT02840955. Registered on 11 July 2016.

**Electronic supplementary material:**

The online version of this article (doi:10.1186/s13063-017-2118-x) contains supplementary material, which is available to authorized users.

## Background

Atopic dermatitis (AD) is a chronic inflammatory skin disease that is associated with reduced quality of life (QoL), primarily due to itchy skin [1–3]. The disease is characterised by reduced skin microbial diversity and overgrowth of *Staphylococcus (S.) aureus,* a bacterium that can aggravate skin inflammation via the production of staphylococcal enterotoxins that stimulate the release of pro-inflammatory cytokines [4–7]. However, the importance of *S. aureus* colonisation in the complex pathogenesis, compared to the other genetic and immunologic factors involved, remains unclear.

Current treatment approaches for AD include topical treatment with emollients and anti-inflammatory therapy with (topical) immunosuppressive agents (corticosteroids and calcineurin inhibitors), according to the international guidelines [8, 9]. Anti-staphylococcal therapy is only recommended in cases of fever or clinically infected skin [8, 9]. Clinical studies that evaluated the added value of anti-staphylococcal therapy in non-infected AD, have shown contradictory results. Bath Hextall et al. performed a systematic review of 26 studies and showed that anti-staphylococcal agents reduced the amount of *S. aureus* on the skin in AD. However, the bacteriological reduction did not translate into a decrease in clinical symptoms [10]. These studies mainly investigated short-term therapies of less than one month duration and comprised small and poor-quality studies. As discontinuation of therapy after a short treatment period can result in quick regrowth of *S.* aureus [11], the results of this systematic review do not necessarily mean that anti-staphylococcal agents do not work. A more recent review by Brüssow et al. summarizes two intervention trials that reported significant reduction in disease severity in non-infected AD after 2 and 3 months of therapy with anti-staphylococcal therapy (bleach baths) [12–14]. We hypothesize that long-term therapy may be needed to reduce *S. aureus* overgrowth and maintain a stable and balanced skin microbial composition. Ultimately, this could result in disease improvement, prevention of AD flares and less need for (topical) immune suppression. However, long-term use of antibiotics can induce bacterial resistance [15], and both the use of antibiotics and dilute bleach baths can cause unnecessary harm to the commensal flora, that is hypothesized to have anti-staphylococcal properties [16].

In the context of the increasing incidence of bacterial resistance, the interest in bacteriophages and their endolysins as antibacterial therapy has been renewed [17]. Staphefekt SA.100 is an engineered chimeric endolysin that specifically lyses the cell membrane of *S. aureus* via endopeptidase and putative amidase activities [18–20]. Long-term application of Staphefekt on the skin, targeting only *S. aureus* and leaving skin commensals unharmed, may improve long-term AD outcomes, such as the number of disease flares, and may reduce the use of topical corticosteroids. Bacterial resistance to Staphefekt or other endolysins has not been observed and could not be induced, which enables us to study the effect of long-term anti-staphylococcal treatment in non-infected AD using this endolysin-based agent [19, 21, 22].

The aim of this randomized controlled trial, the MAAS trial, is to evaluate the effect of 3-month anti-staphylococcal therapy with Staphefekt on the frequency and quantity of topical corticosteroid use, clinical symptoms and QoL in patients with moderate to severe AD. In addition, data on the growth characteristics of the skin microbiome, including *S. aureus*, will be collected, which will gain insight into the role of the microbiome as a factor in the pathophysiology of AD.

## Methods/design

### Design and setting

**Fig. 1:**
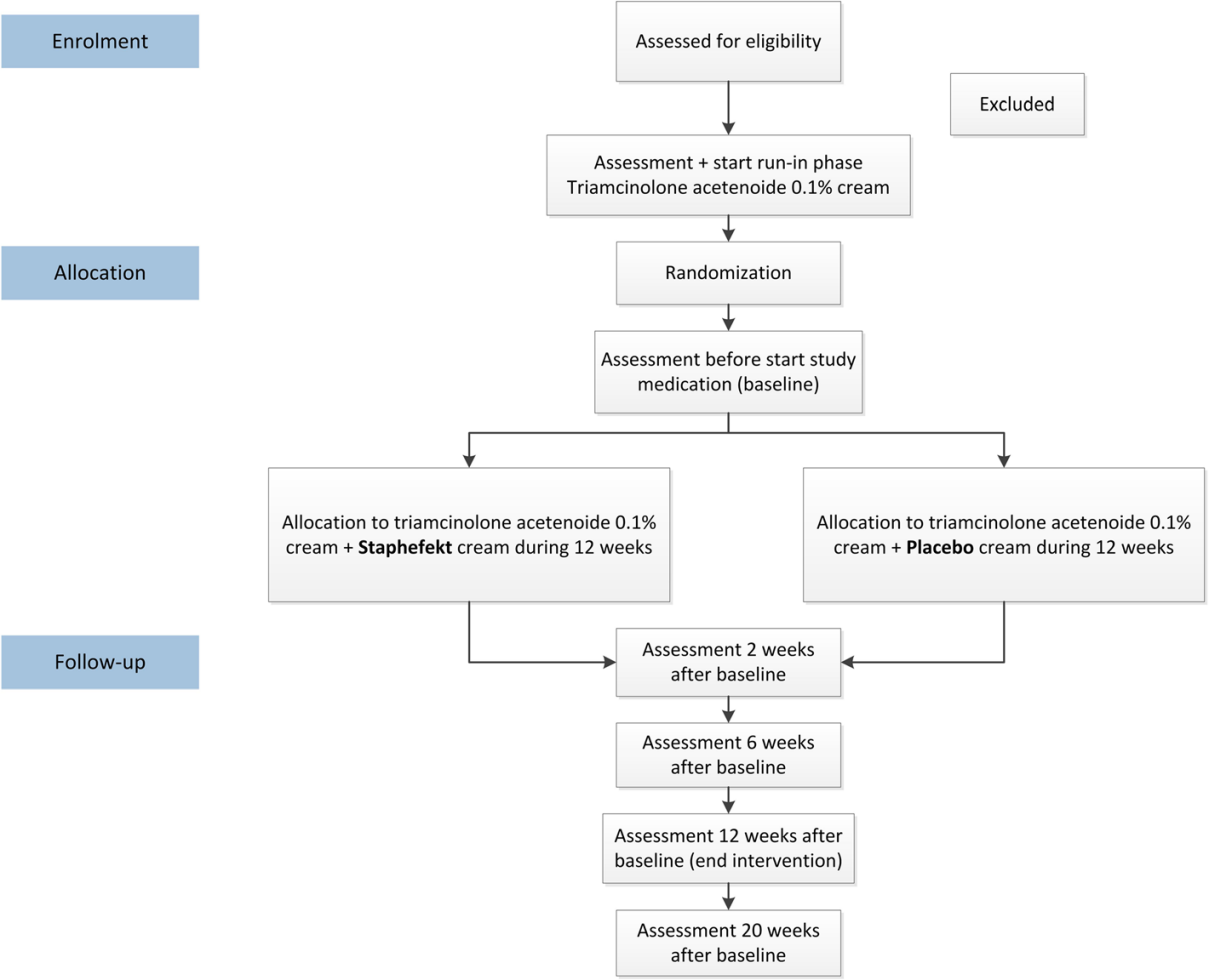
Flowchart of the study design

The MAAS trial (Microbiome in atopic dermatitis during anti-staphylococcal therapy and the effect on steroid use), is a multi-centre, randomized, double-blinded, placebo-controlled superiority trial with a parallel group design (Fig. 1). The study aims to evaluate the effect of Staphefekt on the use of corticosteroids, disease severity, QoL and composition of the microbiome in patients with AD. The study was designed by the Department of Dermatology of the Erasmus MC University Medical Centre Rotterdam and will be executed in collaboration with the Havenziekenhuis Rotterdam. Enrolment and follow-up visits take place at these two locations. Participants who comply with the criteria for inclusion and exclusion will start with a 2-week run-in period to standardize the corticosteroid use with triamcinolone acetonide 0.1% cream. After completion of the run-in phase, participants will be randomized to either treatment with Staphefekt or a placebo for 12 weeks, followed by an 8-week follow-up period. An Eczema Area Severity Index (EASI) above 50 after the run-in phase is a contraindication for further participation. During the course of the study, participants visit the outpatient clinic six times (visit 1 through 6) and data will be collected on corticosteroid use, disease severity, QoL, skin microbiome and adverse events. See Additional file 1 for an overview of the Standard Protocol Items: Recommendations for Interventional Trials (SPIRIT) 2013 checklist items. See Table 2 for the SPIRIT diagram of the trial procedures.

### Ethical considerations

This study follows the Dutch Medical Research Involving Human Subjects Act 1998 (WMO) and the principles of the Helsinki Declaration 2008. All study procedures have been reviewed and approved by the Medical Ethics Committee of the Erasmus MC University Medical Centre Rotterdam, The Netherlands (reference 2016-233). Protocol amendments will be submitted for review at the Medical Ethics Committee.

### Participants

This study will enrol adults (18 years or older) diagnosed with AD according to the UK working party diagnostic criteria for AD [23–25]. Participants are eligible for enrolment if they have a score between 7.1 and 50.0 on the EASI for disease severity. Topical corticosteroids must have been prescribed before enrolment. All patients must be able to read and understand the patient information and provide written informed consent. Patients are not eligible for enrolment if they used: (1) systemic antibiotics or corticosteroids in the 2 months prior to enrolment, (2) oral immunosuppressive agents or UV therapy in the 3 months before enrolment or (3) local antibiotics or Staphefekt (from commercial sources) 1 week before enrolment. Other criteria for exclusion are known contact allergy to any of the components of the study drug (e.g. propylene glycol), clinically infected AD or the existence of other skin condition(s) that could interfere with the assessment of the AD severity.

### Recruitment, inclusion and consent

Participants with AD will be recruited from the dermatology outpatient clinic of the Erasmus MC and the Havenziekenhuis Rotterdam. Furthermore, Dutch dermatologists are informed about the study via the Dutch Trial Network and via scientific conferences. Patients with AD are informed via the patient support group and via online media, such as DermHome (https://www.huidhuis.nl/). In addition, recruiting advertisements will be placed on student forums and in local newspapers. Patients who are interested in participation in the trial can contact the researcher directly via email or phone. After a first screening with regard to the inclusion and exclusion criteria via email or phone, potentially eligible participants receive an information letter and will be invited to the dermatology outpatient clinic to further assess eligibility. Patients who fulfil the inclusion criteria and are willing to participate, will be included in the study after providing written informed consent.

### Sample size

The sample size for this study was calculated based on the primary outcome, namely the difference in mean days per week of corticosteroid use over 12 weeks between the Staphefekt arm and the placebo arm, in patients who are positive for *S. aureus* on the skin at baseline. This is the first study measuring clinical outcomes of Staphefekt in patients with AD. We expect to find a mean topical corticosteroid use of 5 days/week in the placebo group. This was based on a study by Hon et al. that showed decreased use of topical corticosteroids when taking bleach baths, an anti-staphylococcal therapy [26]. Based on the results of this study, we anticipate an effect size of 1.25 day/week reduction in topical corticosteroid use in the Staphefekt arm. A sample size was calculated using the unpaired *t* test to compare means in a superiority trial design. With power of 0.80, alpha of 0.05 and SD of 2.0, 40 patients are needed per treatment arm. Assuming 10% drop out and 90% of the patients being positive for *S. aureus* on the skin lesions, 50 patients will be assigned to each of the two treatment arms.

### Randomization and blinding

The participants are randomly assigned, in a 1:1 fashion to either treatment with Staphefekt or placebo. Stratified block randomization for AD severity is performed to ensure equal distribution of patients with moderate and severe AD over the treatment arms (EASI 7.1–21 and EASI 21.1–50). Randomization is done by an independent biostatistician of the Erasmus MC, using the statistical software package R version 3.2.2. The participants, the researchers and laboratory analyst are blinded to the intervention. The pharmacy manages the randomization list and provides blinded study medication.

### Intervention

After enrolment, all participants start a run-in phase of 2 weeks in which they receive a standardized dosing regimen of topically applied triamcinolone acetonide 0.1% cream (Table 1). After the run-in period, the patient and the researcher evaluate further participation, with very severe AD (EASI >50) as a contraindication for continuation. The run-in period and randomization is followed by a 12-week treatment period and an 8-week follow-up period. During the treatment period, Staphefekt or placebo cream will be applied on the total skin surface twice daily to reach optimal reduction of *S. aureus,* as both lesional and non-lesional skin are often colonised [4]. The Staphefekt endolysin is made available in a cetomacrogol-based cream. The placebo is composed of the same cetomacrogol-based cream, without Staphefekt. During the treatment and follow-up period triamcinolone will be used according to the corticosteroid dosing regimen (Table 1). Measurements and assessments will be performed at enrolment (start of the run-in phase, visit 1), baseline (start treatment with Staphefekt/placebo, visit 2a), 0.5 hours after baseline and 2, 6, 12 and 20 weeks after baseline (visit 2b to 6). Table 2 provides an overview of the measurements per visit. Unless it is in the best interest of the patients (for example in the case of an eczema flare), patients are not allowed to use systemic or topical immunosuppressive medication (including calcineurin inhibitors), antibiotics or antiseptics during the study. Escape medication will be prescribed according to current treatment guidelines and its use will be registered. At the start of the study, patients receive an emollient according to the patient’s preference for use during the course of the study. The use of this emollient will be registered by weighing the tubes at each visit.

**Table 1:** Corticosteroid dosing regimen

	Week 1	Week 2	Week 3	Week 4	Week 5	Week 6	Week 7	Week 8	Week 9
Saturday	X X	X	X	X					
Sunday	X X	X			X	X	X	X	
Monday	X X	X	X	X					
Tuesday	X X	X			X	X			
Wednesday	X X	X	X	X					
Thursday	X X	X			X	X	X	X	
Friday	X X	X	X	X					

Start in week 1 or 2 depending on severity of the atopic dermatitis. If the symptoms allow, reduce the use of corticosteroid cream weekly according to the scheme. Return to week 1 or 2 in the case of an exacerbation. Based on patient assessment

**Table 2:**
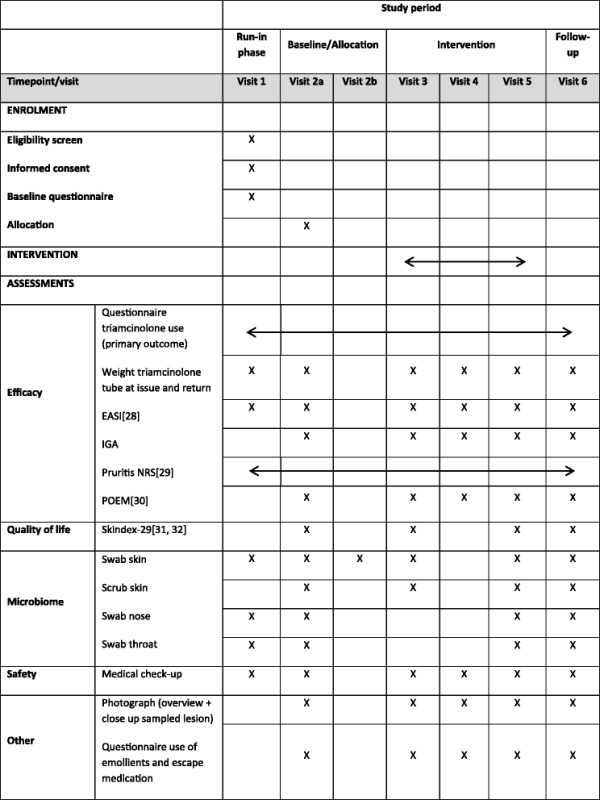
SPIRIT diagram of study procedures [28–32]

Visit 1, enrolment in the trial and start of a 2-week run-in phase; visit 2a, start of the intervention (baseline); visit 2b, 0.5 hours after baseline; visit 3, 2 weeks after baseline; visit 4, 6 weeks after baseline; visit 5, 12 weeks after baseline and end of the intervention; visit 6, follow-up visit 20 weeks after baseline. All visits take place plus or minus 2 days from the indicated timeframe. *EASI* Eczema Area and Severity Index, *IGA* Investigator’s Global Assessment, *Pruritus NRS* Pruritus Numerical Rating Scale, *POEM* Patient Orientated Eczema Measure

### Detailed sample and laboratory procedures

Sampling procedures are based on the “Manual of Procedures” for microbiome sampling of the Human Microbiome Project [27]. All samples are obtained by one of the researchers wearing gloves (sterile for the skin scrub). Sterile Copan 490CE.A swabs are used to sample the skin, nasal cavity and pharynx. Skin samples are taken from lesional skin, preferably located at the antecubital folds or the popliteal fold. The skin surface is swabbed for 30 seconds. The mucosal surfaces of both the anterior nares are gently rubbed, going round the area for 10 seconds. The rear of the oropharynx is swabbed for 5 seconds using a tongue depressor. For the skin scrub sample, a ring with an internal diameter of 4 cm will be placed on the same skin lesion where the swab was collected, but on a non-overlapping area: 1 ml of swab solution (0,85% NaCl, 0.1% bacteriological peptone, 0.1% Tween 80) is pipetted into the ring. After rubbing over the skin with a Copan 480CE swab for 1 minute, the swab solution will be pipetted out of the ring into an Eppendorf tube. The swabs will be sent to the laboratory by mail on the day of collection. A semi-quantitative culture technique and matrix-assisted laser desorption ionization time-of-flight (MALDI-TOFF) for identification of *S. aureus* will be performed. The scrub samples will be stored at –80 °C at the Erasmus MC Rotterdam until 16S rRNA-sequencing and quantitative *S. aureus* analysis.

### Primary and secondary outcomes

The primary outcome of this study is the days per week of corticosteroid use, compared between the Staphefekt and the placebo group over 12 weeks. Patients report their triamcinolone use daily in a secured digital platform, “DermHome”.^1^ Additionally, the use of triamcinolone cream will be measured in grams by weighing the study medication at the time of issue and return (each visit). Secondary outcomes include clinical efficacy and QoL from baseline through week 12 and week 20, change in the microbial composition (including *S. aureus)* and safety. Clinical efficacy is measured using the EASI, the Investigator’s Global Assessment (IGA) and registration of the number of flares [28]. A flare is defined as an exacerbation that requires the need to intensify treatment, from a doctor or patient’s perspective. This implies stronger topical therapy or the need for systemic treatment. A 50% increase in the EASI score compared to baseline is used as an indication to intensify treatment. The Pruritus Numerical Rating Scale (Pruritus NRS) and the Patient Orientated Eczema Measure (POEM) are included as patient-reported efficacy outcomes [29, 30]. QoL is measured using the Skindex-29 [31, 32]. Changes in the microbiome are evaluated by comparing the changes in bacterial composition between the treatment groups, determined by 16S rRNA sequencing of the skin scrub samples. Reduction in *S. aureus* is determined by quantitative PCR (and culture for the comparison between visit 2a and visit 2b). Safety and tolerability is assessed by monitoring the incidence of (serious) adverse device events through the end of the study, evaluated by medical check-ups that include evaluation of vital signs. Reportable adverse events will be reported within the set timelines to the competent authorities. Table 2 gives a detailed overview of the measurements per visit.^1^

### Data collection, monitoring and data analysis

Data collected during the visits are entered in Open Clinica. This data management system allows direct data entry. Data entry is monitored by an independent researcher according to a predefined monitoring plan. Triamcinolone use and itch scores filled in daily by the patients in “DermHome” will be extracted in an SPSS format and combined with the Open Clinica database. Patient confidentiality will be ensured by using identification numbers. Data will be analysed on an intention-to-treat basis. A mixed linear regression model will be used to examine if there is a significant difference in corticosteroid use over 12 weeks between the intervention and the placebo group [34]. This model accounts for repeated measurements in each patient and is valid in the case of missing data. Covariates that could influence the outcome variable will be included in the model. Subgroup analysis will be performed to analyse patients who are positive for *S. aureus* on the skin versus patients that are negative for *S. aureus* before the start of the intervention. Positive patients are defined as having positive cultures both at visit 1 and 2a. Negative patients must have two negative cultures. Patients that have one positive and one negative culture will not be included in the subgroup analysis. Secondary outcomes will also be analysed using a mixed model analysis (linear or logistic according to the type of data). The findings of this study will be published in national and international journals (according CONSORT 2010 Statement) and will be communicated to the relevant patient associations.

## Discussion

The MAAS trial is a randomized, placebo-controlled trial that investigates the effect of 3-month anti-staphylococcal therapy with Staphefekt on topical corticosteroid use, clinical symptoms and QoL in adults with moderate to severe AD. Additionally, data will be collected about the growth characteristics of the skin microbiome, including *S. aureus*. Taking into consideration the current literature on anti-staphylococcal therapy, a study design using a long-term anti-staphylococcal intervention, measuring long-term outcomes was chosen.

Evidence for the clinical efficacy of Staphefekt, registered as a class 1 medical device in Europe, is based on in vitro studies and a case series [18–20]. These in vitro studies showed that Staphefekt kills different strains of *S. aureus* (also methicillin-resistant strains), without harming the commensal flora or inducing bacterial resistance [19]. A case series describes clinical improvement in *S. aureus* related symptoms, such as folliculitis and superinfected dermatitis, and no development of resistance during long-term daily treatment with Staphefekt based on the minimal inhibitory concentrations of the cultured *S. aureus* strains over time [35]. The lack of resistance induction can be expected, as bacterial killing by an endolysin is independent of the involvement of the bacterial metabolism. The co-evolution of bacteriophages and their host bacteria over millions of years, ensures that phage endolysins attack essential bonds in the bacterial cell wall that cannot be adapted by the host [22]. Thereby, the lytic activity of exogenously applied endolysins results in lysis of the target cells within seconds, restricting the possibility to adapt and develop resistance. Furthermore, attacking several bonds of the bacterial wall simultaneously by the use of more than one enzymatically active domain in the Staphefekt molecule, makes resistance even less likely to develop [18].

Because of the proteinaceous nature of endolysins, immunogenicity can be of concern. The literature shows the possibility of the formation of non-neutralizing antibodies against lysins other than Staphefekt [22]. In a study in which the presence of anti-Staphefekt IgG was evaluated in serum from 21 Staphefekt-naive healthy human donors, pre-existent IgG antibodies recognizing Staphefekt epitopes were detected in all the donors (unpublished data). This can be explained, as humans are exposed daily to *S. aureus* and therefore to bacteriophages and their lysins. However, Staphefekt is a large protein molecule (>50 kDa), making penetration through the skin and mucosa and subsequent antibody reactions unlikely [36].

Calculation of the sample size for this study was hampered as no information was available about the effect of Staphefekt on corticosteroid use and clinical efficacy in AD. Therefore, the study should be considered as hypothesis generating, giving insight into effect sizes and distributions of clinical outcomes. Our expected effect size was based on a study of Hon et al. of the effect of bleach on corticosteroid use in AD. We chose a slightly higher effect size, because we expect the effect of Staphefekt that specifically targets *S. aureus* to be more efficacious than bleach. We consider this effect size, a reduction in corticosteroid use of more than 1 day a week over 12 weeks, as clinically relevant because of the (low) risk of side effects and a general reluctance of patients to use corticosteroids, resulting in poor compliance and a lack of treatment efficacy [37, 38].

No consensus has been reached yet on a standardized outcome for long-term AD control, the primary goal of our study. The Harmonising Outcome Measures for Eczema (HOME) initiative reached consensus on the use of EASI and POEM as doctor-based and patient-based measures of AD severity, both of which are included as secondary outcomes in this trial [39]. According to the authors of HOME, measures of long-term control could include time to flare and the use of rescue medicine [39]. Next to corticosteroid use, both these study outcomes were included in this study as secondary parameters.

In conclusion, this study will evaluate the effects of 3-month targeted anti-staphylococcal therapy with Staphefekt in moderate to severe AD. The lack of resistance induction allows long-term treatment with this anti-staphylococcal agent. This study will provide the first data on the use of anti-staphylococcal therapy with Staphefekt in AD and may provide new insights into the role of *S. aureus* in the pathophysiology of AD.

### Trial status

The first patient was included in the study in July 2016. Patient recruitment is currently ongoing and the recruitment is expected to be completed by August 2017.

## Additional file

Additional file 1: SPIRIT 2013 checklist: recommended items to address in a clinical trial protocol and related documents. (DOC 121 kb)

## Abbreviations

AD: Atopic dermatitis
EASI: Eczema Area and Severity index
IGA: Investigator’s global assessment
kDa: KiloDalton
MAAS trial: Microbiome in atopic dermatitis during anti-staphylococcal therapy and the effect on steroid use trial
POEM: Patient Orientated Eczema Measure
Pruritus NRS: Pruritus Numerical Rating Scale
QoL: Quality of life
*S. aureus*: *Staphylococcus aureus*
SPIRIT: Standard Protocol Items: Recommendations for Interventional Trials

## Footnotes

1 “DermHome” is a secured digital treatment and research platform, developed in collaboration with Patient 1 BV, Almere [33]. The platform provides a user-friendly individual account that allows patients to report their pruritus score and triamcinolone use daily. Thereby the platform provides digital information about the study, including the use of the study medication, and an option to contact the researcher and to upload photos in case of questions. After every visit the researcher can make notes in the digital file about findings, agreements and future appointments.
